# Dual Genetic Diagnosis of Prader–Willi Syndrome and TMC1-Related Severe Congenital Hearing Loss: Diagnostic Challenges and Cochlear Implant Outcomes

**DOI:** 10.3390/diagnostics16020300

**Published:** 2026-01-17

**Authors:** Pinelopi Samara, Michail Athanasopoulos, Evangelia Koudoumnaki, Nikolaos Markatos, Ioannis Athanasopoulos

**Affiliations:** 1 Children’s Oncology Hospital “Marianna V. Vardinoyannis-ELPIDA”, 115 27 Athens, Greece; 2 Department of Otology, Audiology, and Pediatric Neurotology, Pediatric Center of Athens, 151 25 Athens, Greece; athamich@med.uoa.gr (M.A.); markatoshearing@gmail.com (N.M.); dr.iathanasopoulos@gmail.com (I.A.)

**Keywords:** Prader–Willi syndrome, TMC1, sensorineural hearing loss, deafness, cochlear implant, case report

## Abstract

**Background and Clinical Significance:** Prader–Willi syndrome (PWS) is an imprinting disorder not typically associated with severe congenital sensorineural hearing loss (SNHL). When profound SNHL is present in an infant with a known syndrome, an independent monogenic etiology should be considered. We report the first molecularly confirmed case of PWS co-occurring with biallelic pathogenic TMC1 variants causing congenital SNHL, outlining diagnostic challenges, cochlear implant (CI) outcomes, and implications for blended phenotypes. **Case Presentation:** A male infant with PWS due to a paternal 15q11.2–q13 deletion failed newborn hearing screening. Diagnostic auditory brainstem response and auditory steady-state response confirmed bilateral severe-to-profound SNHL. Temporal bone CT/MRI were normal. Comprehensive genetic testing identified compound heterozygous TMC1 variants consistent with autosomal recessive DFNB7/11 hearing loss, plus two variants of uncertain significance in *SERPINB6* and *EPS8L2*. Sequential bilateral cochlear implantation was performed (left ear at 14 months, right at 20 months), followed by auditory–verbal therapy. Over four years, the child showed steady improvements in hearing and early-speech development. **Conclusions:** Early genomic evaluation is essential when clinical features appear atypical for a known syndrome. Identifying TMC1-related deafness enabled timely cochlear implantation and measurable gains. This case highlights that severe congenital SNHL in a syndromic infant may reflect a distinct monogenic disorder rather than phenotypic expansion of the primary syndrome, emphasizing the importance of recognizing blended phenotypes to guide precision-care strategies in rare disorders.

## 1. Introduction

Prader–Willi syndrome (PWS) is a rare and complex genetic imprinting disorder, with an estimated incidence ranging from 1:15,000 to 1:30,000 live births worldwide [1]. The syndrome arises from the loss of paternally expressed genes in the chromosome 15q11.2-q13 region, most commonly due to a de novo paternal deletion (accounting for approximately 65–75% of cases), maternal uniparental disomy (UPD, 20–30%), or defects in the imprinting center (2–5%) that disrupt normal epigenetic regulation [2]. These distinct genetic mechanisms result in a deficiency in several critical genes, including those encoding small nucleolar RNAs and regulatory proteins, which collectively contribute to the broad spectrum of clinical manifestations.

Phenotypically, PWS is characterized in infancy by severe hypotonia, poor feeding, weak suck, and failure to thrive, often necessitating specialized nutritional and supportive interventions [3]. As affected children grow, the syndrome evolves to include developmental delays, intellectual disability of variable severity, distinct craniofacial features, behavioral challenges, hypothalamic dysfunction, endocrine abnormalities such as growth hormone deficiency, central adrenal insufficiency, hypogonadism, and a transition to hyperphagia, which frequently results in severe obesity if not carefully managed [4,5,6].

Beyond these hallmark features, PWS exerts multisystem effects, impacting neurocognitive, metabolic, endocrine, autonomic, and sensory domains. Increasing evidence has highlighted the presence of sensory disturbances as a consistent component of the syndrome, reflecting its complex neurodevelopmental underpinnings [7]. Recent work demonstrates that children with PWS exhibit measurable differences in postural control and sensory integration, particularly in how visual, somatosensory, and vestibular inputs are weighted for balance, compared to children without PWS [8]. Among sensory modalities, ocular abnormalities are particularly prevalent, with studies reporting high rates of strabismus, refractive errors, amblyopia, and, more rarely, structural anomalies such as foveal hypoplasia or choroidal hypopigmentation [9,10,11]. In addition to visual disturbances, PWS is associated with alterations in other sensory systems, including atypical nociception with reduced pain sensitivity, impaired thermoregulation, vestibular dysfunction affecting balance and motor coordination, and olfactory processing differences that may contribute to hyperphagia and abnormal food-seeking behaviors [12,13,14,15,16].

Collectively, these findings underscore the conceptualization of PWS as a multisensory neurodevelopmental disorder, in which abnormal sensory integration and processing are integral to the phenotype. The wide-ranging involvement of multiple organ systems, coupled with variability in sensory and neurobehavioral manifestations, highlights both the clinical heterogeneity of PWS and the importance of comprehensive, individualized, and multidisciplinary management strategies across the lifespan, including early detection and targeted interventions to optimize functional outcomes.

However, auditory impairment is not a recognized feature of the PWS phenotype; reports of hearing loss are rare and anecdotal. The International PWS Organization (IPWSO) includes audiological assessments in clinical guidelines primarily due to parental reports of auditory hypersensitivity or concerns about delayed language development rather than as part of a recognized risk for congenital hearing loss [17]. Only two published reports have documented congenital or early-onset sensorineural hearing loss (SNHL) in PWS: one presenting unilateral profound SNHL of unknown cause [18], and another involving a maternal UPD case with a distinct genomic diagnosis (biallelic STRC–CATSPER2 deletion, causing deafness–infertility syndrome) unrelated to PWS pathophysiology [19]. These cases underscore that when significant hearing loss occurs in a child with PWS, it warrants investigation for an independent genetic etiology.

The TMC1 (transmembrane channel-like 1) gene, located on chromosome 9q12, is a well-established cause of autosomal recessive (DFNB7/11) and, less commonly, autosomal dominant (DFNA36) SNHL [20,21]. TMC1 encodes a transmembrane protein expressed in cochlear hair cells that is essential for mechanotransduction, converting sound-induced mechanical stimuli into electrical signals [22]. Biallelic pathogenic variants in TMC1 typically lead to prelingual, severe-to-profound congenital SNHL, often with a stable hearing phenotype, whereas heterozygous dominant variants are associated with progressive postlingual hearing loss [23]. Functional studies have demonstrated that TMC1 deficiency disrupts hair-cell stereocilia structure and ion channel function, confirming its central role in cochlear mechanosensory transduction [24]. Identification of pathogenic TMC1 variants therefore provides a clear molecular diagnosis in infants presenting with congenital deafness. Importantly, studies also indicate that cochlear implant outcomes are robust in TMC1-related deafness [25], reflecting preserved auditory nerve function. Moreover, recent genotyped cohorts, including TMC1 patients, show excellent short- and long-term outcomes, with age at implantation and auditory experience influencing performance [26].

Here, we report the first known case of an infant with genetically confirmed PWS and concomitant compound heterozygous pathogenic TMC1 variants causing severe-to-profound congenital SNHL. This case documents the coexistence of two independent molecular diagnoses, demonstrates how genomic testing in syndromic infants can reveal blended phenotypes, and provides early cochlear implant outcomes in a child with dual diagnoses.

## 2. Case Presentation

### 2.1. Clinical Overview

A 3-month-old male infant was referred for audiological evaluation after failure of newborn transient-evoked otoacoustic emissions (TEOAE) screening. Pregnancy and delivery were uneventful. The infant was born at term via spontaneous vaginal delivery with a birth weight of 3.1 kg. He exhibited marked neonatal hypotonia, weak suck, and feeding difficulties, requiring modified feeding strategies. Genetic testing shortly after birth confirmed PWS due to paternal deletion of the 15q11.2–q13 region.

The family history was negative for hearing loss, congenital anomalies, neurodevelopmental disorders, or known genetic conditions. Neither parent reported personal or familial hearing impairment. The infant was receiving nutritional and endocrine monitoring as part of standard PWS management. Motor development was delayed but improving with early physiotherapy.

### 2.2. Audiological Assessment

**Figure 1:**
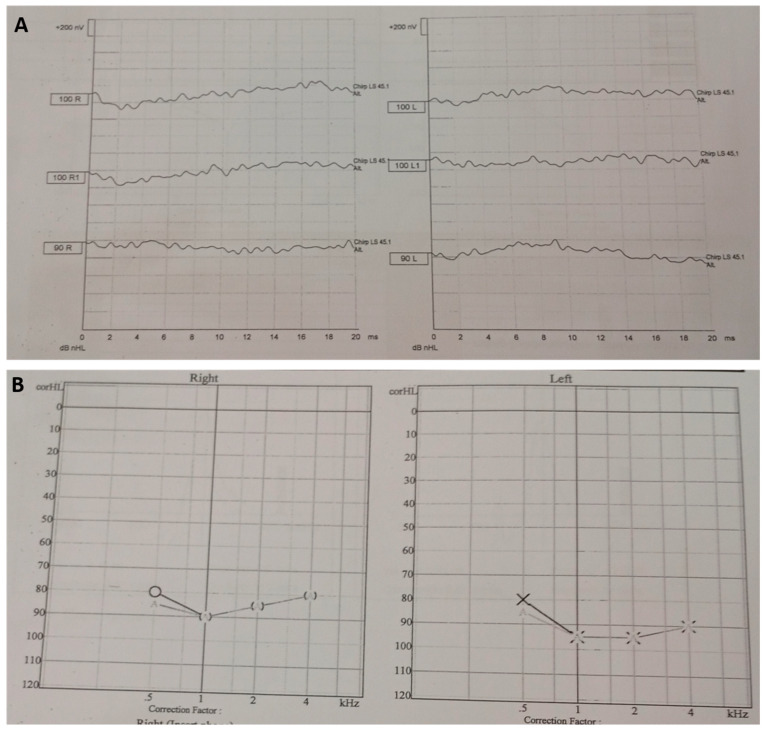
Audiological assessment at 3 months of age. (**A**) Auditory brainstem response (ABR): No identifiable waveforms were observed bilaterally, consistent with severe-to-profound sensorineural hearing loss (SNHL). (**B**) Auditory steady-state response (ASSR): No responses were detected at any frequency tested (0.5, 1, 2, 4 kHz) or intensity, confirming bilateral severe-to-profound SNHL.

Initial screening with TEOAE revealed that responses were absent bilaterally. Tympanometry revealed type A curves, indicating normal middle-ear function without effusion. Diagnostic auditory brainstem response (ABR) and auditory steady-state response (ASSR) at 3 months confirmed bilateral severe-to-profound SNHL. ABR results demonstrated no replicable waveforms at maximum output levels, consistent with severe-to-profound SNHL. ASSR thresholds were near the equipment limits across frequencies in both ears, confirming profound bilateral SNHL (Figure 1). These results prompted expanded evaluation due to the unexpected presence of congenital deafness in a child with PWS, where hearing loss is not normally associated.

### 2.3. Imaging

**Figure 2:**
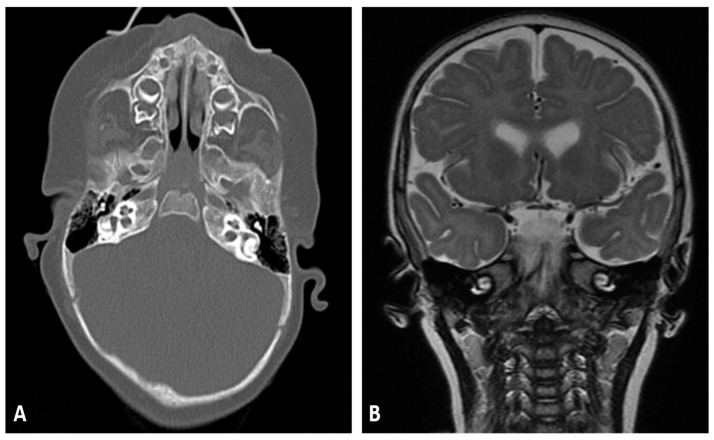
High-resolution temporal bone imaging. (**A**) Axial CT scan showing normal cochlear, vestibular, and middle-ear anatomy without bony abnormalities or malformations. (**B**) MRI of the internal auditory canal demonstrating normal cochlear nerve morphology and intact vestibulocochlear structures, with no evidence of nerve deficiency or other structural anomalies.

High-resolution computed tomography (CT) of the temporal bones demonstrated normal cochlear and vestibular anatomy, patent semicircular canals, normal middle-ear structures, and no evidence of bony dysplasia. Magnetic resonance imaging (MRI) of the internal auditory canals confirmed normal morphology of the cochlear nerves and vestibulocochlear structures (Figure 2). The absence of structural abnormalities supported a likely genetic cause for the hearing loss.

### 2.4. Genetic Analysis

Molecular analysis of hearing loss was conducted using a comprehensive gene panel targeting known hearing-loss-associated genes. Total genomic DNA was extracted from peripheral blood samples. Sequencing was carried out using Illumina technology, generating 150-base-pair reads with an average coverage of ≥100× across targeted regions. Raw image data from the sequencing run were processed using Illumina BaseSpace Sequence Hub (version 3.8; Illumina, San Diego, CA, USA) and the resulting sequences were converted to FASTQ format. Bioinformatic analysis included quality control, alignment using Burrows–Wheeler Aligner (BWA-MEM; version 0.7.12), and variant calling with GATK algorithms as implemented in the Sentieon DNAseq® pipeline (Sentieon, San Jose, CA, USA). Additionally, deletions, duplications, and copy number variations (CNVs) were assessed in genes associated with hearing loss.

This analysis identified two heterozygous variants in TMC1:

- c.1114G>A, p.(Val372Met)—classified as pathogenic
- c.332G>A, p.(Trp111)—classified as likely pathogenic

Parental segregation analysis revealed that the variants were inherited in trans, confirming autosomal recessive DFNB7/11. Two additional variants of uncertain significance (VUS) were detected in genes implicated in hair-cell biology: SERPINB6 c.983C>T, p.(Thr328Met) and EPS8L2 c.616G>T, p.(Ala206Ser) [27,28]. Although these genes play functional roles in cochlear hair-cell protection and stereocilia organization, respectively, current evidence is insufficient to establish pathogenicity or to attribute a significant contribution to the observed phenotype. Variant classification was performed following the American College of Medical Genetics and Genomics (ACMG) guidelines. All molecular findings, including inheritance pattern and predicted functional impact, are summarized in Table 1.

### 2.5. Management

The patient was initially fitted with conventional hearing aids to provide maximal auditory stimulation and to assess the presence and functional utility of any residual hearing. The hearing aids were maintained until the completion of the necessary administrative procedures and scheduling for cochlear implantation. Despite consistent use during this period, no measurable functional benefit was observed. Following comprehensive multidisciplinary evaluation involving pediatric audiology, otolaryngology, neurodevelopmental specialists, and speech–language pathology, the decision was made to proceed with sequential bilateral cochlear implantation.

The left cochlear implant was performed at 14 months of age, followed by implantation of the right ear at 20 months. The sequential approach was deliberately selected to allow close monitoring of potential vestibular impacts and postoperative balance function in the context of PWS, which is frequently associated with hypotonia, delayed motor milestones, and subtle vestibular dysfunction. This staged strategy enabled the clinical team to evaluate tolerance to unilateral implantation and assess early auditory and vestibular responses before proceeding with contralateral surgery.

Both surgical procedures were uneventful. Intraoperative neural response telemetry demonstrated appropriate functioning of all electrode contacts, confirming effective auditory nerve stimulation. Postoperative imaging with CT verified full insertion of both electrode arrays in optimal position, with no evidence of intra-cochlear trauma or malposition. The patient tolerated general anesthesia and postoperative recovery without complications, supporting the feasibility of cochlear implantation in infants with complex syndromic neurodevelopmental disorders.

### 2.6. Rehabilitation

Following activation of the cochlear implants, the child commenced weekly auditory-verbal therapy under the supervision of a specialized pediatric cochlear implant team. Therapy goals, including sound detection, discrimination, identification, and comprehension, were adapted to the patient’s dual diagnosis. Potential cognitive difficulties, hypotonia, and sensory integration differences associated with PWS were considered, and the child required extended time to process auditory information and execute verbal instructions. Strategies such as deliberate pauses, repetition, acoustic highlighting, and rephrasing were systematically applied to support learning and optimize engagement.

Structured qualitative assessments were conducted using the Clinical Evaluation of Language Fundamentals (CELF), the Derbyshire Screening Test, and the STASS. Results were interpreted descriptively because these tests are not standardized for the Greek population. Additional custom-developed protocols were used to qualitatively assess auditory discrimination, auditory memory, and sound recognition abilities.

During the initial phase of rehabilitation, the child demonstrated measurable improvements across multiple domains of auditory perception, speech, and language development. He exhibited enhanced detection and localization of environmental sounds, consistent orientation to auditory cues, and emergent speech-like vocalizations, indicating functional engagement with the auditory environment. The child became capable of detecting the presence or absence of sounds, responding to complex auditory stimuli, and associating specific sounds with corresponding objects and contexts. Emerging auditory recognition of family members and familiar social cues was observed, along with the ability to execute simple verbal commands. Reaction times for comprehension and task execution were occasionally prolonged, reflecting the broader neurodevelopmental profile of PWS. Speech perception and production progressed steadily, with the patient responding appropriately to social and interactive prompts, demonstrating integration of auditory input with motor and communicative outputs.

At a follow-up evaluation at 4 years, further consolidation and expansion of auditory, language, and speech skills were noted. Auditory abilities were well-established: he consistently responded to environmental and speech sounds, accurately discriminated objects across lexical categories, and demonstrated functional understanding of verbs and object use. He reliably comprehended basic questions, familiar phrases, and simple commands without visual cues, and understood fundamental spatial, quantitative, and qualitative concepts. Auditory memory for single items was established, with emerging two-item memory skills. Standardized tests administered at this stage included the Action Picture Test and the Word Finding Test, which are normed for the Greek population. On the Action Picture Test, descriptive adequacy corresponded to the 40th percentile and grammatical adequacy to the 30th percentile based on chronological age. On the Word Finding Test, lexical retrieval performance corresponded to the 30th percentile. Assessment based on auditory age was not possible, as the tests are standardized for children aged 4–8 years. Nevertheless, performance would be expected to be higher if auditory age norms were applicable.

Overall, expressive language showed significant progress. The patient produced utterances of up to three words, demonstrated emerging grammatical awareness including correct subject-verb agreement, and utilized imperative and past tense forms as well as negation. Articulation and oral-motor skills remained areas of ongoing development, with difficulties in lip closure affecting bilabial and labiodental consonants, as well as occasional phoneme substitutions and cluster simplifications.

### 2.7. Additional Clinical Notes

The child’s endocrine and nutritional management remained consistent with PWS best-practice guidelines. Ophthalmologic examination was unremarkable. No additional neurological abnormalities were identified. The family received genetic counseling focusing on the dual diagnosis and implications for developmental and educational planning.

**Figure 3:**
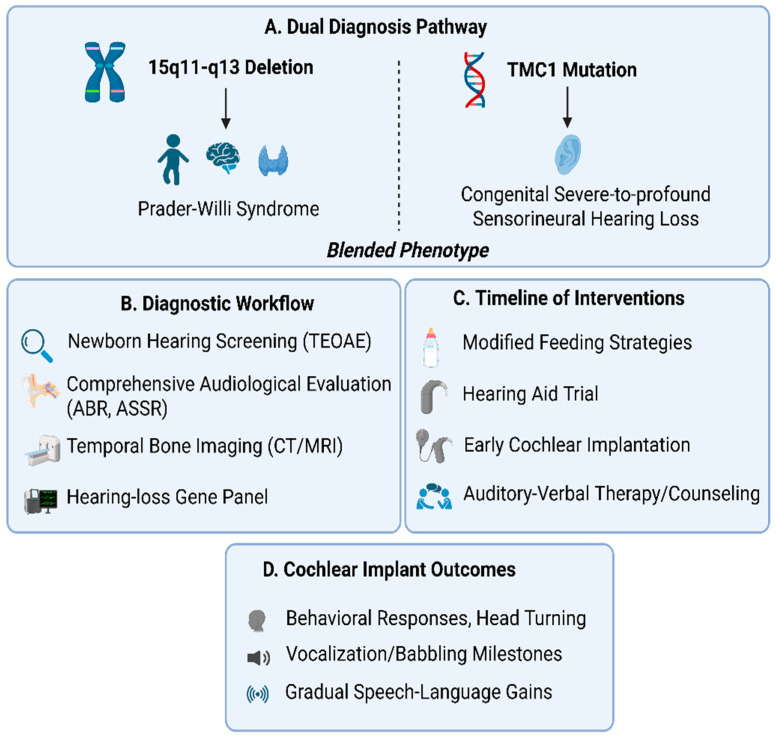
Comprehensive Management Overview of an Infant with Dual Genetic Diagnosis. (**A**) Dual diagnosis pathway illustrating the identification of Prader–Willi syndrome and biallelic TMC1 variants. (**B**) Diagnostic workflow for syndromic infants failing newborn hearing screening, including audiological, radiological, and genetic assessments. (**C**) Timeline of clinical interventions, including sequential cochlear implantation. (**D**) Cochlear implant outcomes over four years, highlighting improvements in auditory detection, sound localization, and early speech–language milestones. Created with BioRender.com (accessed on 4 December 2025). ABR—Auditory Brainstem Response; ASSR—Auditory Steady-State Response; TEOAE—Transient Evoked Otoacoustic Emissions.

Figure 3 depicts the integrated workflow—from dual genetic diagnosis to cochlear implantation and developmental follow-up—in the infant with PWS- and TMC1-related congenital hearing loss, providing a framework that can be applied to the clinical care of other infants with syndromic presentations and multidimensional needs.

## 3. Discussion

This report describes, to the best of our knowledge, the first case of genetically confirmed TMC1-related congenital severe-to-profound SNHL in a patient with PWS, representing a “blended phenotype” in which a classical imprinting syndrome is compounded by an independent monogenic sensory disorder. The co-occurrence underscores a critical diagnostic principle: even in individuals with a well-established genetic syndrome, the emergence of phenotypic features that are atypical or uncharacteristic of that syndrome—especially when they are severe or congenital—should invoke expanded genetic evaluation rather than be reflexively attributed to the primary diagnosis.

From a mechanistic perspective, TMC1 is now widely accepted as a principal component of the mechano-electrical transducer channel in cochlear hair cells, the specialized sensory receptors responsible for converting mechanical stimuli at the stereocilia into electrical signals that enable hearing. Mice lacking functional TMC1 (especially in combination with TMC2) are deaf; expression of exogenous TMC1 or TMC2 in such double-knockout hair cells rescues mechanotransduction [29]. Additionally, human and mouse TMC1 mutations linked to non-syndromic deafness alter mechano-electrical transducer channel properties—notably reducing Ca^2+^ permeability, lowering resting open-probability under physiological conditions, and in some cases reducing channel conductance or surface expression, ultimately leading to hair-cell degeneration and sensorineural deafness [30,31].

In our patient, the biallelic TMC1 variants provide a biologically plausible, hair-cell-based explanation for the profound congenital SNHL, independent of PWS pathophysiology. Thus, this is not simply a coincidental co-occurrence at the clinical level, but a genuine dual genetic diagnosis with a well-demonstrated molecular basis.

By contrast, although PWS is associated with multisystem features including neurodevelopmental delay, hypotonia, endocrine dysfunction, and sensory processing abnormalities (e.g., altered olfaction, somatosensory differences, motor delay) [7], congenital severe SNHL is not a recognized or common aspect of PWS, and reports of hearing impairment are anecdotal. Only two published reports have documented congenital or early-onset SNHL in PWS: one presenting unilateral profound SNHL of unknown cause [18], and another involving a maternal UPD case with a distinct genomic diagnosis (biallelic STRC–CATSPER2 deletion, causing deafness–infertility syndrome) unrelated to PWS pathophysiology [19]. The rare prior reports of hearing impairment in PWS described either unilateral or moderate-to-severe SNHL, and in both cases, the hearing loss may have been coincidental rather than intrinsic to the syndrome. In this context, attributing the profound congenital SNHL of our patient solely to PWS lacks biological and pathogenetic justification. The TMC1 findings therefore represent a second, independent disease rather than an expansion of the PWS phenotype.

This distinction carries significant clinical and management implications. First, recognizing a dual diagnosis enables more accurate prognostic counseling: families can be informed that the hearing loss likely stems from a well-characterized monogenic etiology (TMC1), with predictable course, rather than an unpredictable variable manifestation of PWS. Second, because TMC1-related deafness arises from defective mechanotransduction at the hair-cell level, early intervention—notably prompt cochlear implantation—remains rational, even in a complex syndromic background. Early auditory stimulation is pivotal in these children, as timely exposure to sound engages neuroplastic mechanisms during critical developmental windows, thereby conferring the greatest attainable potential for language acquisition and broader cognitive development. In our patient, despite the neurodevelopmental vulnerabilities inherent to PWS, cochlear implant surgery, anesthesia, and postoperative rehabilitation were well-tolerated, and early auditory progress was observed. This suggests that cochlear implantation is feasible, safe, and beneficial in syndromic patients when managed within a multidisciplinary team.

Nevertheless, some caveats and limitations warrant acknowledgement. While TMC1 (and its paralog TMC2) are strongly implicated in hair-cell mechanotransduction, the exact molecular architecture of the channel remains incompletely resolved; interactions with accessory proteins (e.g., tip-link components, membrane lipids, trafficking molecules) may modulate channel function, conductance, Ca^2+^ permeability, and hair-cell survival. In addition, in the context of a complex epigenetic disorder such as PWS—which involves imprinting dysregulation and wide-ranging neurodevelopmental consequences—one cannot fully exclude the possibility that genetic, epigenetic, or environmental modifiers influenced the auditory phenotype, though no evidence for such modifiers is currently available. Finally, this is a single case, and caution is required in generalizing these findings or inferring prevalence of dual diagnoses in PWS populations.

Beyond PWS, the implications of this case could extend to other rare neurological, neurodevelopmental, or multisystem genetic disorders. Emerging precision genomics literature emphasizes that individuals with complex neurodevelopmental phenotypes—particularly when accompanied by additional neurological or systemic features—benefit from comprehensive genomic evaluation, which can uncover clinically actionable diagnoses beyond the primary syndrome [32]. In practice, expanded testing in syndromes such as Angelman, Rett, and Smith–Magenis has begun to reveal additional variants or co-occurring conditions that explain atypical features or complications in those patients. In such contexts, the emergence of atypical features—for example, unexpected hearing impairment, vision deficits, or metabolic disturbances—should prompt clinicians to consider additional genetic or monogenic diagnoses rather than assume they represent novel features of the primary syndrome.

As genomic sequencing becomes increasingly accessible, systematic screening of syndromic cohorts (especially those with atypical or “non-canonical” features) may reveal a greater prevalence of “blended phenotypes.” Documenting such cases can refine genotype–phenotype correlations across rare disorders, inform personalized management, guide genetic counseling, and ultimately support the development of genotype-driven therapies.

## 4. Conclusions

This case demonstrates that TMC1-related congenital SNHL can coexist with PWS, producing a blended phenotype. The identification of biallelic pathogenic TMC1 variants provides a mechanistic, hair-cell-based explanation for the SNHL, independent of PWS pathophysiology. As this report concerns a single patient, caution is warranted in generalizing these findings to other individuals with PWS or similar syndromes. For infants with PWS who present with unexplained hearing impairment—especially severe or congenital SNHL—expanded genetic testing (such as gene panels or even whole-exome sequencing) should be strongly considered. Early audiological assessment and timely cochlear implantation remain beneficial, even in complex syndromic contexts, when managed by a multidisciplinary team. More broadly, systematic recognition of dual diagnoses in rare neurological and multisystem syndromes will improve genotype–phenotype correlations, optimize patient management, and support advance toward precision medicine in neurogenetics.

## Figures and Tables

**Table 1:** Summary of molecular genetic findings in the patient.

Gene/Region	Variant (HGVS)	Zygosity	Pathogenicity	Notes
15q11.2–q13	Loss of paternally expressed genes (deletion)	Heterozygous	Pathogenic	Consistent with molecular diagnosis of Prader–Willi syndrome
*TMC1*	c.1114G>A, p.(Val372Met)	Heterozygous	Pathogenic	Well-established cause of autosomal recessive DFNB7/11 hearing loss
*TMC1*	c.332G>A, p.(Trp111)	Heterozygous	Likely pathogenic	Associated with congenital severe-to-profound SNHL; ACMG LP
*SERPINB6*	c.983C>T, p.(Thr328Met)	Heterozygous	VUS	Involved in cochlear hair-cell protection; variant not previously linked to hearing loss
*EPS8L2*	c.616G>T, p.(Ala206Ser)	Heterozygous	VUS	Important for stereocilia structure; clinical significance unknown

VUS, Variant of Uncertain Significance; SNHL, Sensorineural Hearing Loss; ACMG, American College of Medical Genetics and Genomics.

## Data Availability

All data supporting the findings of this study are contained within the article.

## Abbreviations

ABR	Auditory Brainstem Response
ASSR	Auditory Steady-State Response
PWS	Prader–Willi Syndrome
SNHL	Sensorineural Hearing Loss
TEOAE	Transient-Evoked Otoacoustic Emissions
TMC1	Transmembrane Channel-Like 1
UPD	Uniparental Disomy
VUS	Variant of Uncertain Significance
